# Clinical and demographic profile of cancer patients in a consultation-liaison psychiatric service

**DOI:** 10.1590/S1516-31802003000300005

**Published:** 2003-05-01

**Authors:** Vanessa de Albuquerque Citero, Luiz Antonio Nogueira-Martins, Maria Teresa Lourenço, Sergio Baxter Andreoli

**Keywords:** Referral and consultation, Consultations, Psychiatry, Mental Health Services, Evoluation Studies, Oncology Service, Cancer care Facilities, Cancer, Epidemiology, Serviço hospitalar de oncologia, Consulta, Psiquiatria, Serviços de saúde mental, Estudos de avaliação, Cancêr, Epidemiologia

## Abstract

**CONTEXT:**

An almost 50% prevalence of psychiatric disorders among cancer patients has prompted a series of studies on consultation-liaison psychiatry. Nonetheless, there are few reports on the epidemiological factors involving comorbidity between cancer and psychiatric disorders.

**OBJECTIVE:**

To evaluate the epidemiological profile of cancer inpatients referred to the consultation-liaison psychiatric service in an oncology hospital during its first year of activity.

**TYPE OF STUDY:**

Descriptive study.

**SETTING:**

Tertiary-care teaching hospital.

**PARTICIPANTS:**

319 patients referred 412 times to the consultation-liaison psychiatry service.

**PROCEDURES:**

From August 97 to July 98, an appraisal was made of data on all admissions registered at the Hospital do Câncer, and also all referrals registered at the consultation-liaison psychiatry service.

**MAIN MEASUREMENTS:**

The demographics and patients’ clinical data, the type and flow of the request, and the evaluation conducted by the service were analyzed and comparisons with the hospital data were made. The distribution of the number of referrals was used to construct a profile of patients who had repeatedly used the service.

**RESULTS:**

Psychiatric diagnoses were found in 59% of the cases. Forty-three percent of these required medication, 18.3% needed psychotherapy, 22.1% family intervention and 20.5% guidance from the staff. Over 22.8% of the consultations were reevaluations, mainly involving younger male patients with worst prognoses. These patients required lengthier and more elaborate intervention, and had higher prevalence of depressive and behavioral disorders.

**CONCLUSION:**

A younger and mainly male population of non-surgical oncological cases was referred to the consultation-liaison psychiatric service during its first year of activity. The psychiatric disorder prevalence was higher than expected, and consisted predominantly of mood disorders. We detected a priority group, namely the reevaluated patients, who deserved special attention throughout the psychiatric interventions.

## RESUMO

**CONTEXTO:**

Diversos estudos sobre interconsulta psiquiátrica mostram uma prevalência de cerca de 50% de comorbidade psiquiátrica em pacientes com câncer. No entanto, poucos registros sobre os fatores epidemiológicos que propiciam o estabelecimento desta comorbidade foram encontrados.

**OBJETIVO:**

Avaliar o perfil epidemiológico dos pacientes com câncer, internados num hospital oncológico e encaminhados para o serviço de interconsulta psiquiátrica, durante o primeiro ano de seu funcionamento.

**TIPO DE ESTUDO:**

Estudo descritivo.

**LOCAL:**

Hospital terciário de ensino privado.

**PARTICIPANTES:**

319 pacientes encaminhados por 412 pedidos de interconsulta psiquiátrica.

**PROCEDIMENTOS:**

Todas as internações realizadas no Hospital do Câncer e todas as interconsultas solicitadas no período de agosto de 97 a julho de 98 foram registradas e analisadas.

**VARIÁVEIS ESTUDADAS:**

Os dados demográficos e clínicos do paciente, os dados sobre o pedido e o fluxo do atendimento, condutas e diagnósticos do serviço de interconsulta foram analisados e comparados com os dados das internações. A distribuição do número de atendimentos foi utilizada para construir o perfil do paciente que repetidamente era encaminhado para o serviço.

**RESULTADOS:**

59% dos pacientes apresentavam transtorno psiquiátrico; 43% destes necessitaram de medicação psicotrópica, 18,3% de psicoterapia, 22,1% de intervenção sobre a família e 20,5% de orientação para a equipe de saúde. Quase 23% das solicitações foram para reavaliações, sendo a maioria delas para pacientes do sexo masculino, mais jovens e de pior prognóstico. As reavaliações se caracterizaram por atendimentos mais prolongados, por intervenção mais complexa e por maior prevalência de transtorno depressivo. Os pacientes reavaliados constituíram um grupo problema, necessitando da elaboração de atenção psiquiátrica especial.

**CONCLUSÃO:**

Uma população de pacientes oncológicos mais jovem, predominantemente do sexo masculino e de setores não-cirúrgicos foi encaminhada ao serviço de interconsulta no seu primeiro ano de funcionamento. A prevalência de transtornos psiquiátricos foi maior do que a esperada, predominantemente os quadros de transtorno do humor. Os pacientes reavaliados constituíram um grupo prioritário na demanda do serviço, necessitando de especial atenção no desenvolvimento de intervenções psiquiátricas.

## INTRODUCTION

Structured psychiatric referral services in general hospitals have been demanded because of either higher prevalence of psychiatric disorders or longer lengths of stay. In such settings, patients suffering from co-morbidity or psychiatric complications imply a 50% cost increase.^1,2^ This is also valid for oncological hospitals and services, because psychiatric disorders prevail in approximately 50% of the patients.^3,4^

Several studies have evaluated the prevalence of psychiatric disorders among cancer patients. Derogatis et al.^3^ showed that, from a psychopathological standpoint, 53% of cancer patients had no psychiatric disorders according to the Diagnostic and Statistical Manual Mental Disorders version III (DSM III) criteria. In the other 47%, a pathological clinical picture is present: 85% anxiety and/or depressive disorders, 8% cerebral organic disorders and 7% personality disorders. A review study^5^ on cancer patients referred to the consultation-liaison psychiatry service showed depression prevalence ranging from 9% to 58%. In another study, adjustment disorders were found in 11% to 21% of the cancer patients admitted to the hospital.^6^ Delirium is a common mental disorder in general hospitals, and in cancer patients the expected prevalence rate is 25%, whereas in terminal cancer patients the rate rises to 85%.^7^ Psychoses and cognitive impairment have been shown to play a major role in delays in compliance or non-compliance with oncological treatment among such patients.^8^

In a recent review on psycho-oncology, Citero^9^ pointed out that cancer does, in fact, facilitate an increase in psychiatric morbidity. This is especially true in depressive and adjustment disorders, although epidemiological reports are scarce.

With regard to epidemiological studies, no information has emerged regarding the characteristics of cancer patients requiring evaluation or follow-up. Our main objective was therefore to present a study on the epidemiological profile of patients referred to the consultation-liaison psychiatry service at the Treatment and Research Center of the Hospital do Câncer AC Camargo, during its first year of activity.

## METHOD

From August 1997 to July 1998, an appraisal was made of data on all admissions registered at the Hospital do Câncer, and also all referrals registered at the consultation-liaison psychiatry service (n = 412). The Hospital do C âncer is a 200-bed referral center in Sao Paulo, Brazil.

The consultation-liaison psychiatry service can operate 24 hours a day, whenever physicians, nurses and other staff from the hospital's other departments request the evaluation of an inpatient. The standard daily routine of the 3 consultation-liaison psychiatrists includes receiving either written or oral requests, visiting the wards, collecting information from the consultant doctor, evaluating the patient's records and evaluating the patient and his/her caregiver. Following initial evaluation, the patient, caregiver and staff are presented with a diagnosis and, if necessary, a therapy proposal plan.

The consultation file record is designed for data to be distributed into 4 groups, as follows : I. patient demographics, including previous psychiatric treatment and present diagnosis of cancer, classified according to the International Classification of Diseases 10^th^ revision criteria (ICD-10); II. data on the request for consultation; III. data on the consultation flow; IV. data on diagnosis and management at the consultation-liaison psychiatry service, classified according to the ICD-10 criteria.

Data analysis was performed by direct comparison of the rates, distributed into different categories of variables, and not by comparisons using statistical methods, because probability sampling was not employed. The rates corresponding to the distribution of patients evaluated according to the studied variables were used in the report. The distribution of the number of referrals was used to construct a profile of patients who had repeatedly used the service. The Tables presented do not consider the loss of data, which was insignificant (less than 5%) for all the evaluated variables and groups.

## RESULTS:

The referrals to the consultation-liaison psychiatry service corresponded to 5% of the patients admitted to the hospital. The demographics of the two populations (consultation-liaison patients versus total hospital inpatients) had different features. In Table 1, it can be seen that, in the total hospital inpatients group, there was a much higher rate of female patients (56.3%) and those in older age groups (71.8% over 30 years old). The patient distribution for the consultation-liaison psychiatry service group, on the other hand, revealed that 50.5% of patients were male (χ^2^ = 8.6; p < 0.001), and 63.6% were over 30 years old (χ^2^ = 17.9; p < 0.001).

**Table 1:** Frequency of hospital admissions and consultation-liaison psychiatry service evaluations, according to sex, age and medical division

		Hospital admissions		Consultation-liaison psychiatry service evaluation	
		N	%	N	%
Sex*	Female	4540	56.3	199	48.3
	Male	3520	43.6	208	50.5
	No data	10	0.1	5	1.2
Age*	0 to 18 years old	1716	21.3	90	21.8
	19 to 30 years old	540	6.7	46	11.2
	31 to 59 years old	3250	40.3	161	39.1
	60 years old and over	2551	31.5	101	24.5
	No data	13	0.2	14	3.4
Medical division*	Clinical Oncology	1083	13.4	180	43.7
	Pediatric	1248	15.5	96	23.3
	Surgical area	4380	54.3	74	18.0
	Other	1358	16.8	59	14.3
	No data	1	0.0	3	0.7
**Total**		**8070**	**100.0**	**412**	**100.0**

* *p < 0.001.*

The original admission ward, however, showed the greatest difference in relation to inpatient distribution at the hospital (Table 1). Over 50% of the hospital's inpatients were admitted to surgical units (Gynecology, Mastology, Head and Neck, Chest, Pelvis and Abdomen Surgery). However, the greatest percentage of patients referred to the consultation-liaison psychiatry service (43.7%) was originally from the Clinical Oncology Unit. The pediatric unit also registered a higher rate of patient referrals to the consultation-liaison service (23.3%), in comparison with the 15.5% of hospital admissions that this unit represented. The difference between the medical divisions was statistically significant (χ^2^ = 358.6; p < 0.001).

The referral patients’ demographic characteristics showed that these were individuals with a low education level (55% having barely completed grade school), residing in the São Paulo region (67%), married (59%) and inactive for periods of over 4 months since referral or dedicated to economically inactive occupations such as retired pensioners, housewives or students (84%). The gender distribution showed that the female patients were from an older age group than the male patients, less educated, less economically active, and presented higher rates of legal separation or widowhood and previous psychiatric disorders.

These 412 evaluations corresponded to 319 patients, signifying that 93 evaluations were reevaluations of the same patient. It implies that 23% of the total number of referrals was for reevaluations. Although multiple hospital admissions are a very frequent event in oncological centers, 22% of the patients (70 out of 319) were referred twice or more, at different admission times. Among the 93 reevaluation referrals, the patients were predominantly male, younger (32% under 30 years of age), unmarried, inactive and from outside of the São Paulo region.

Almost 74% of the patients referred did not have a history of previous psychiatric disorder. The neoplastic diagnoses were as follows: 23.4% in lymphatic and hematopoietic tissue (predominantly men), 18% in female genitals (including breast cancer), 11.3% in the respiratory tract and 10.3% in the lips or oral cavity. The latter two were predominantly found in male patients. Most of the evaluations (64%) were requested by the physicians, mainly for psychiatric evaluation and management (69%). The reevaluation requests showed higher incidence of previous histories of psychiatric disorders (mostly in women, Table 2) and higher frequencies of patients with undefined sites and lymphatic/hematopoietic tissue neoplasms (both of which are predominant in male patients) and patients with bone and cartilage neoplasms (predominant in female patients). The reevaluations followed the same pattern presented in all other evaluations, namely, the greatest frequency was among pediatric male patients, with requests being made for reasons other than evaluation and management, by non-medical professionals (Table 2).

**Table 2:** Number of evaluations and number of patients evaluated by the consultation-liaison psychiatry service, distributed according to previous contact with the psychiatric service, type of neoplasm, professional requests and reason for request, divided by sex

		Number of evaluations				Number of patients evaluated			
		Male		Female		Male		Female	
		N	%	N	%	N	%	N	%
**Previous psychiatric disorder**	Yes	62	29.1	81	40.7	25	14.6	41	27.7
	No	140	65.7	108	54.3	135	78.9	98	66.2
	No data	11	5.2	10	5.0	11	6.5	9	6.1
**Neoplasm type**	Lymphatic hematopoietic tissue	67	31.5	33	16.6	49	28.7	24	16.2
	Female genitals			66	33.2			54	36.5
	Respiratory tract	29	13.6	14	7.0	23	13.5	11	7.4
	Lips, oral cavity	27	12.7	10	5.0	24	14.0	7	4.7
	Bones/Cartilage	17	8.0	20	10.1	14	8.2	10	6.6
	Unidentified site	18	8.5	13	6.5	9	5.3	9	6.1
	Gastrointestinal	14	6.6	8	4.0	12	7.0	5	3.4
	Eyes, central nervous system	5	2.3	10	5.0	5	2.9	8	5.5
	Others	25	11.7	17	8.5	24	14.0	12	8.1
	No data	11	5.1	8	4.1	11	6.4	8	5.5
**Professional who requested**	From physicians	138	64.8	110	55.2	113	66.1	88	59.5
	From other staff	66	31.0	85	42.7	51	29.8	57	38.5
	No data	9	4.2	4	2.1	7	4.1	3	2.0
**Reasons for request**	Evaluation and approach	141	66.2	119	59.7	122	71.3	94	63.5
	Other requests	64	30.0	77	38.6	42	24.6	51	34.5
	No data	8	3.8	3	1.7	7	4.1	3	2.0
**Total**		**213**	**100.0**	**199**	**100.0**	**171**	**100.0**	**148**	**100.0**

With regard to the actions taken by the consultation-liaison psychiatry service in order to fulfill such requests (Table 3), it was noted that in 90.3% of the cases, the patients were consulted within 24 hours. In 51.3% of the cases, the consultations were continued across a 5-day period (i.e. 1 to 4 visits were made by the psychiatrist). In reevaluations, the duration of the psychiatric consultation period upon first admission was longer (more than 11 days). Disorders were diagnosed in almost 60% of the patients (more frequently in men) (Table 3), as follows: mood disorders (30.5%, predominantly mild and major depressive disorders); neurotic or somatoform disorders (17.4%, of which 14% were predominantly adjustment disorders); and organic and somatic disorders (7.3%, of which 4% were predominantly delirium disorders, mostly in male patients).

**Table 3:** Number of evaluations and number of patients evaluated by the consultation-liaison psychiatry service, distributed according to the time taken to initiate, duration of the evaluations and psychiatric diagnosis, divided by sex

		Number of evaluations				Number of patients evaluated			
		Male		Female		Male		Female	
		N	%	N	%	N	%	N	%
**Time until**	From 1 to 2 days	182	87.5	185	93.0	146	88.0	137	92.3
**^1st^ evaluation**	From 3 to 7 days	24	11.5	12	6.0	18	10.8	9	6.1
	No data	2	1.0	2	1,0	2	1.2	2	1.6
**Duration of evaluation**	From 1 to 5 days	107	51.4	102	51.2	87	52.4	78	52.7
	From 6 to 68 days	81	38.9	80	40.2	63	38.0	55	37.2
	No data	20	9.7	17	8.6	16	9.6	15	10.1
**Diagnosis of psychiatric disorders**	None	77	37.2	82	41.2	62	37.3	64	43.2
	Mood	63	30.3	61	30.7	45	27.1	44	29.7
	Neurotic & somatoform	42	20.1	29	14.6	37	22.3	22	14.9
	Organic & somatic	16	7.6	14	7.0	14	8.5	8	5.4
	Others	10	4.8	10	5.0	8	4.8	7	4.7
	No data			3	1.5			3	2.1
**Total**		**208**	**100.0**	**199**	**100.0**	**166**	**100.0**	**148**	**100.0**

Among the types of intervention made by the consultation-liaison (Table 4), it was noted that 43% of the patients referred received some form of medication, 18% were submitted to brief psychotherapy and 22% required family intervention. In these 3 categories there was a higher prevalence of male patients. The interventions for guidance of staff members (20.5%) occurred mostly in relation to female patients. Inpatient follow-up was interrupted in 64.3% of the cases because of hospital discharge, and 26.9% were discharged by the consultation-liaison service. Upon hospital discharge, only 30% of the patients were referred to our outpatient psychiatric follow-up. For the remaining 67.4%, this did not occur, either because it was not considered necessary, or because treatment had been interrupted at the time of clinical discharge. The reevaluations displayed characteristics differing from the population generally seen by the service. In these cases, the need for intervention was more frequent and mood (mostly among male patients) and organic/somatic disorders were the most prevalent diagnoses.

**Table 4:** Number of evaluations and number of patients evaluated by the consultation-liaison psychiatry service, distributed according to the types of approach established and referrals, divided by sex

		Number of evaluations				Number of patients evaluated			
		Male		Female		Male		Female	
		N	%	N	%	N	%	N	%
**Medication approach**	Yes	98	46.0	83	41.7	79	46.2	57	38.5
	No	110	51.6	114	57.3	87	50.9	89	60.1
	No data	5	2.3	2	1.0	5	2.9	2	1.4
**Brief psychotherapy**	Yes	41	19.2	34	17.1	32	18.7	25	16.9
	No	167	78.4	163	81.9	134	78.4	121	81.8
	No data	5	2.3	2	1.0	5	2.9	2	1.4
**Family evaluation**	Yes	54	25.4	39	19.6	40	23.4	29	19.6
	No	154	72.3	158	79.4	126	73.7	117	79.1
	No data	5	2.3	2	1.0	5	2.9	2	1.4
**Guidance for staff members**	Yes	40	18.8	53	26.6	30	17.5	34	23.0
	No	168	78.9	144	72.4	136	79.5	112	75.7
	No data	5	2.3	2	1.0	5	2.9	2	1.4
**Discharge**	From consultation-liaison	49	23.0	56	28.2	40	23.4	44	29.7
	From hospital	140	65.7	127	63.8	109	63.7	93	62.8
	Death	18	8.5	16	8.0	16	9.4	11	7.5
	No data	6	2.8			6	3.5		
**Referral**	None	134	62.9	136	68.3	108	63.2	103	69.6
	Outpatient clinic	68	31.9	60	30.2	53	31.0	43	29.0
	Another service	5	2.4	3	1.5	4	2.3	2	1.4
	No data	6	2.8			6	3.5		
**Total**		**213**	**100.0**	**199**	**100.0**	**171**	**100.0**	**148**	**100.0**

## DISCUSSION

The consultation-liaison psychiatry service has become a well-known service within the hospital's dynamics, as evidenced by the large number of referrals. The authors did not find any data on other consultation-liaison psychiatry services in cancer hospitals in Brazil, but in comparison with other general hospitals, the percentage of referrals to our service was higher than expected. A review of American studies^10^ showed that the referral rate to consultation-liaison psychiatry services in most general hospitals in the United States is around 1%, even though the prevalence of psychiatric disorders range from 30% to 60% in such services. We found the same situation described in several of the Brazilian studies, indicating an incidence of 1% to 2.5%, which may vary depending on the type of service or increase in accordance with the recognition of the importance of the service.^11^

By being relatively quick in providing evaluations for almost all patients (within 24 hours) the consultation-liaison psychiatry service fulfilled the need for this type of emergency assistance. We noted that in such cases, the severity of the problem called for a quick response, thus contributing towards future referrals of the same person. The set of interventions aimed at serving the patient, the patient's family and the staff members in question, and this was achieved by creating a flexible service, as would be expected of an effective consultation-liaison psychiatric service.^12,13^ Not only were 100% of the patients evaluated with psychiatric follow-up, with or without medication and/or psychotherapy, but also more than 20% of families and staff members were given guidance.

Evaluations were mainly requested for young, male patients admitted to the clinical units. During the evaluation period, patients with this kind of profile were in fact more difficult for staff members to handle. Younger patients demanded more from staff members because their families required special attention, because of their great concern regarding the disease. Consequently, the high rate of requests from the pediatric unit came as no surprise, especially those requiring the evaluation of depression and behavioral changes in the child, along with the psychological follow-up for the parents. Although there are very few reports in the literature on such evaluations in pediatrics, a study conducted at the pediatric unit of the Memorial Sloan-Kettering Cancer Center^14^ found that requests were mostly made for the evaluation of behavioral changes and depression, and there were less requests for advising the patient's family.

The low education level among the population studied came as no surprise, because 23% of this population were pediatric patients and this contributes to a decrease in the average number of school years attended. The same reasons apply to the high number of patients who have been inactive for over 4 months.

Patients with a history of previous psychiatric disorders were assessed according to the reports given either by the patients themselves or by members of their families. Only one in every 4 patients evaluated at our service had a history of previous psychiatric disorders, and thus most of the cancer patients were being submitted to their first consultation by a psychiatrist. This raises the question of whether or not cancer could be a trigger for psychiatric symptoms. Although only 25% of the population in the present study reported previous psychiatric disorders, this is significant, since other studies have shown a correlation between previous psychiatric disorders and cancer.^15^

The high prevalence of requests for psychopathological evaluation indicates that the professionals had considerable familiarity with psychiatric terms. These terms, on the other hand, would sometimes be misused, by calling a given problem a psychiatric symptom. This would therefore mask other situations, such as dealing with a given patient or family member, or dealing with an unsatisfactory course of treatment.

Since the population of the present study was not selected by random sampling, but from a preselected population of emotionally disturbed patients, we expected a higher prevalence of psychiatric diagnoses in our analysis than has been described in the literature.^3^ The confirmation of this was the 60% prevalence rate for psychiatric disorders.^5,6^ It can therefore be assumed that the staff member requesting the patient evaluation had good discernment in detecting psychiatric disorders and referring them correctly to the consultation-liaison psychiatry service.

Although there was a high number of patients discharged from hospital during our evaluation, the close working ties between the consultation-liaison psychiatry service and the outpatient clinic allowed them to continue with the treatment. For the same reason, most of the patients were discharged from hospital without a formal referral from the consultation-liaison service. On the other hand, these patients were instructed by their own physicians to report to the psychiatric outpatient clinic, upon being discharged from hospital.

In this analysis, special mention needs to be made of the reevaluation reports, which represented 23% of the total turnover at our service. Such evaluations were intended for a given portion of the population referred to the consultation-liaison psychiatry service and considered as a problem group. These patients caused the mobilization of staff at the Clinical Oncology unit, due to the need for psychiatric and/or psychological care. The reason for this was either because of the presence of psychopathological symptoms, or because staff members were having difficulty in striking up a relationship with the patient, thus requiring the more elaborate involvement of the consultation-liaison psychiatrists. This problem group had accumulated several factors with bad prognosis: the patients were younger, suffered mostly from leukemia, lymphomas and Ewing's sarcoma (all of these in the more aggressive form, as expected for this age group) and did not live in the region (coming without the family, which was one more stress factor). The evaluations were longer, more complex, required several types of approach and most of the patients died. Not only were there depressive disorders, but also behavioral problems. The referrals came on account of suicide risk, need for family intervention (usually problems with the patient's mother), or requests for psychological follow-up.

## CONCLUSION

A younger and mainly male non-surgical oncological population was referred to the consultation-liaison psychiatric service during its first year of activity. The psychiatric disorder prevalence was higher than expected, and consisted predominantly of mood disorders. Better knowledge of the epidemiological profile of Brazilian cancer patients referred to consultation-liaison psychiatry services has become essential for developing intervention models for mental health applied to our realities. It is crucial to emphasize that reevaluated patients were a priority group, deserving special attention throughout the development of the specific psychiatric interventions.
